# Pluripotent Stem Cells in Disease Modeling and Drug Discovery for Myotonic Dystrophy Type 1

**DOI:** 10.3390/cells12040571

**Published:** 2023-02-10

**Authors:** Noémie Bérenger-Currias, Cécile Martinat, Sandrine Baghdoyan

**Affiliations:** Institut National de la Santé et de la Recherche Médicale (INSERM/UEPS) UMR 861, Université d’Evry/ Paris Saclay, I-Stem, AFM 91100 Corbeil-Essonnes, France

**Keywords:** myotonic dystrophy type 1, human pluripotent stem cells, disease modeling, drug screening

## Abstract

Myotonic dystrophy type 1 (DM1) is a progressive multisystemic disease caused by the expansion of a CTG repeat tract within the 3′ untranslated region (3′ UTR) of the dystrophia myotonica protein kinase gene (*DMPK*). Although DM1 is considered to be the most frequent myopathy of genetic origin in adults, DM1 patients exhibit a vast diversity of symptoms, affecting many different organs. Up until now, different in vitro models from patients’ derived cells have largely contributed to the current understanding of DM1. Most of those studies have focused on muscle physiopathology. However, regarding the multisystemic aspect of DM1, there is still a crucial need for relevant cellular models to cover the whole complexity of the disease and open up options for new therapeutic approaches. This review discusses how human pluripotent stem cell–based models significantly contributed to DM1 mechanism decoding, and how they provided new therapeutic strategies that led to actual phase III clinical trials.

## 1. Introduction

Myotonic dystrophy type 1 (DM1 or Steinert’s disease, OMIM#160900), is the most common type of muscular dystrophy in adults, with an estimated prevalence of one in every 2000 births [1]. Clinically, DM1 is extremely variable, with various symptoms affecting multiple organs, including progressive muscle wasting, muscle hyperexcitability (myotonia), cardiac arrythmias, insulin resistance, gastrointestinal dysfunctions, posterior iridescent cataracts, and cognitive, intellectual, or behavioral impairments. Congenital myotonic dystrophy corresponds to the most severe form of the disease, starting at birth and considered as a life-threatening condition [2,3].

DM1 is an autosomal dominant disease belonging to a large group of disorders associated with the expansions of simple repetitive elements within specific genes [4]. In the specific case of DM1, this is an expanded trinucleotide (CTG) repeat in the 3′ untranslated region of the DM1 protein kinase gene (*DMPK*). The number of those repeats globally correlates with the symptoms’ severity, as well as with the age of onset. In the most severe congenital forms, the number of repeats can reach over 1000, while in non-affected individuals, the number of repeats is around 35 [5]. The number of CTG repeats commonly increases in successive generations, thus the children of DM1 patients will likely have more severe phenotypes than their parents [6]. The number of CTG repeats is usually unstable within the patient’s lifetime, and tends to increase in some body tissues [7]. Several studies have suggested that one of the main pathological mechanisms for DM1 was dependent on an RNA gain-of-function of the mutated *DMPK* transcripts [8,9,10,11]. Indeed, it has been observed in muscular biopsies of DM1 patients that the resulting CUG-containing RNAs (CUG-RNA) accumulate in the cell nucleus into microscopically visible structures called foci [12,13]. These ribonuclear foci, considered to be the most prominent histopathological hallmark of the disease, sequester crucial RNA binding factors. Typically, members of the muscleblind-like (MBNL) family are trapped in those structures, thus preventing their normal functions in the regulation of alternative splicing [10,11,14]. Some results also suggest that the stress responses triggered by the toxic CUG-RNA cause the up-regulation of the MBNL antagonists CELF1 [8,15,16,17]. Both MBNL and CELF1 proteins are required for normal splicing regulation during development, and their imbalance in DM1 results in the abnormal presence of fetal alternative splicing in the tissues of adults patients [14,18,19,20]. So far, hundreds of genes affected by those mis-splicings have been described in DM1 patients, with a couple that have been associated with clinical symptoms such as muscle weakness, myotonia, and insulin resistance [21,22,23,24,25,26]. More recently, different findings have suggested that the pathogenic mechanisms of DM1 may be more complex, involving epigenetic changes, dysregulated gene expression and translation efficiency, antisense transcripts, micro-RNA deregulation, and RNA translation (for recent reviews see [27,28]).

Despite the undeniable advances in the understanding of the DM1 disease, there is currently no curative treatment available to halt or slow down the disease progression. Medical care is thus limited for now to symptomatic treatment, and therapeutic advances are urgently needed [28,29,30,31]. Up until now, several animal models and cell-based assays have been developed to study the physiopathology and evaluate the potential therapeutics [32,33,34,35,36]. Most of the cell-based assays developed so far have included patient-derived fibroblasts or skeletal muscle cells, also named myoblasts [12,37,38,39,40,41,42]. Although studies using these approaches have been crucial for our current understanding of the physiopathology of DM1, each has its limitations. In particular, the multisystemic aspect of DM1 still requires the development of relevant cellular models to cover all the complexities of this disease. Over the past 20 years, the development and the increased accessibility of human pluripotent stem cells have displayed an exceptional potential for human disease modeling and drug testing (for recent reviews see [43,44]). In the present review, we will discuss the development of human pluripotent stem cell-based models for DM1 and their contribution to the advances in myotonic dystrophy research.

## 2. Conventional Cellular Models for DM1

The first cellular models developed to study DM1 largely consisted of cultures of primary dermal fibroblasts and skeletal muscle myoblasts. Those cells are isolated from the patient’s biopsy and conserve the physiological characteristics of their origin tissue environment. Primary fibroblasts were widely used due to their reasonable accessibility in patients. However, primary myoblasts are obviously more relevant for neuromuscular disorders due to their potential to initiate an in vitro myogenic differentiation and fusion into multinucleated myotubes. The insights provided by the use of these patient-derived cells have been crucial for the understanding of the pathomechanisms involved in DM1. The characterization of these models revealed the nuclear retention of the mutant DMPK transcripts while these transcripts are correctly spliced and polyadenylated [12,45]. This phenomenon causes their aggregation within discrete ribonuclear foci, as well as a reduction in the DMPK protein levels [12,45,46,47]. The observation of an aberrant nuclear accumulation of the RNA-binding protein, CELF1 (also called CUG-BP), in cells derived from DM1 patients, led to the suggestion that DM1 is caused by an RNA gain-of-function mechanism [8,48]. CELF1 is a splicing regulatory factor that binds to single-stranded UG motifs, and its up-regulation has been associated with the abnormal splicing of downstream targets [49]. Those observations confirmed the hypothesis of an RNA gain-of-function mechanism, altering cellular function, including the alternative splicing of various genes. This idea was later reinforced by the identification of RNA-binding proteins that bind specifically to CUG repeats and are sequestered by the hairpin structures of the retained CUG-containing DMKP mRNA [10]. In particular, the members of the MBNL RNA-binding proteins family (MBNL1, MBNL2, and MBNL3) are trapped within the nuclear RNA foci in DM1 patient cells [11]. MBNL proteins are splicing regulatory factors involved in the developmental switch between the fetal and adult isoforms of many transcripts. Consequently, the imbalance of MBNL proteins due to their nuclear sequestration of CUG-RNA results in the alternative splicing misregulations of several genes in DM1 [20]. Thus far, MBNL related mis-splicings have been established as a central cause of the disease, since several of the genes affected were connected to DM1 phenotypic features [21,22,23,24,25,26].

Thus, the use of primary cellular models for DM1 has been crucial for the identification and the reproduction of several DM1-associated molecular features. In particular, it highlighted the nuclear aggregation of mutant mRNAs that sequester the MBNL1 protein, leading to subsequent alternative splicing defects [47,50,51,52,53]. Among these primary cellular models, fibroblasts are still widely used both to identify new pathogenic mechanisms, and validate or identify new potential therapeutic compounds. As an example, an increase in autophagic flux, in association with an alteration of the endosomal–lysosomal pathway, has been recently identified in DM1 primary fibroblasts in comparison with control cells [54]. The identification of these pathogenic mechanisms consequently opens new perspectives in terms of therapeutic development [55,56,57]. Illustrating this, a comparative transcriptomic analysis of primary fibroblasts isolated from healthy donors and DM1 patients revealed an impaired cell cycle progression and DNA damage response in DM1, leading to the identification of the therapeutic potential of senolytic compounds for DM1 [56].

Despite these advances, several limitations challenge the use of primary cell cultures. The first concern is the accessibility and availability of patients’ biopsies, especially muscle biopsies. Another point to consider is the fact that primary cells in culture rapidly undergo senescence and can be maintained only for a limited time in vitro. This phenomena has been shown to be even more pronounced in DM1 cells compared to control cells, as the former shows a reduced proliferative capacity due to a premature entry into replicative senescence, thus limiting the extension of their culture [58,59].

To circumvent these limitations, several studies report the generation of immortalized cell lines derived from DM1 primary fibroblasts, trans-differentiated fibroblasts, and myoblasts [37,38,39,40,60,61]. In theory, those lines can be kept in culture indefinitely. They are obtained by maintaining the telomere length through the re-expression of the catalytic subunit of the human telomerase (hTERT), coupled with the overexpression of CDK4 that inhibits p16 activity [60,62]. In addition, clonal selection during this immortalization process leads to the generation of homogeneous cell cultures, providing more consistent and reproducible results [37]. Overall, those immortalized cell lines retain the molecular hallmarks of DM1 related to the toxic RNA gain-of-function mechanism, including nuclear foci and splicing defects. The benefit of their long-term culture potential thus represents a valuable biological resource for high-throughput screening approaches [38,61]. However, the effects of the viral transduction for the genomic integration of hTERT and CDK4 transgenes in immortalized cells are not fully characterized and may alter disease or tissue characteristics.

Along with these DM1 immortalized cell lines, cell models engineered to express exogenous CTG repeats have been widely developed. These models rely on the transient or stable expression of the CTG repeats in the 3′ UTR of a truncated *DMPK* gene in human or murine cell lines, such as HeLa, HEK, or C2C12 cells [19,24,63,64,65,66,67,68]. These models reproduce several DM1-associated features, such as the nuclear foci, the sequestration of MBNL proteins, and the splicing defects. However, in the absence of the complete *DMPK* genomic context, the expression of these CTG constructs is usually controlled by a strong promoter such as CMV promoter, which induces an overexpression greater than the endogenous *DMPK* promoter does [24,64,65,66]. In addition, due to the difficulties of cloning long DNA strands made of CTG repeats [69,70], most of these studies have been using a construct coding for a sequence of 960 CTG repeats, interrupted multiple times by non-repeat short sequences. If interrupted repeats can be found in some patients, their relation to physiopathology and their symptom severity are not well defined yet.

**Figure 1:**
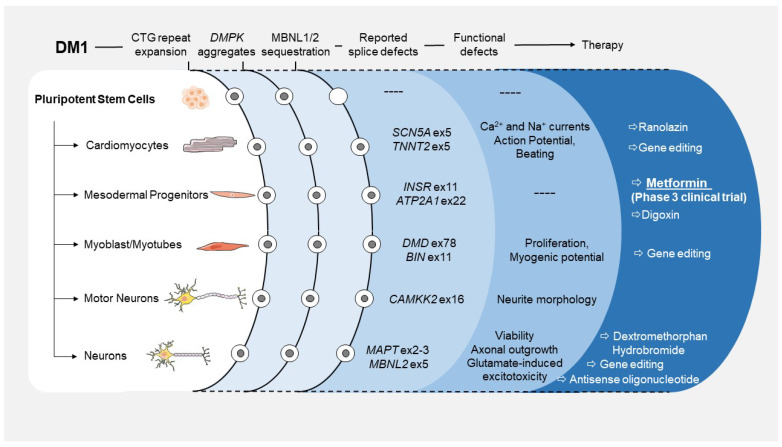
Summary of studies and developments made in in vitro models based on the use of DM1 human pluripotent stem cells and their derivatives.

In conclusion, since the first descriptions of the causal mutation of DM1 30 years ago, different cell models have largely contributed to the knowledge of DM1, mainly on its muscle physiopathology. However, as DM1 is a multisystemic disorder, and many tissues and cell types are affected by this disease. Consequently, there is still a crucial need for relevant cellular models to cover all the complexities of DM1. This understanding of the disease’s complexity is crucial to the identification of new therapeutic approaches. In addition, the comprehension of the physiopathology mechanisms is also limited, due to the difficulty in obtaining material that reflects early stages of the disease (Figure 1). Recent advances in human pluripotent stem cell biology have opened up the possibility of generating new relevant cellular models for DM1. These new tools allowed priceless access to cells impossible to obtain from biopsies, bringing new insight on the disease. In the context of DM1, those studies even led to the identification of new therapeutic approaches actually under clinical investigation.

## 3. The First DM1 Human Pluripotent Stem Cell-Based Models to Study CTG Repeat Instability

In the early 2000s, DM1 was one of the first examples of monogenic diseases to be captured using the physiological source of pluripotent stem cells, namely human embryonic stem cells (hESC) [71]. Thanks to the popularization of preimplantation genetic diagnosis (PGD), the elimination of affected embryos provided a valuable source for hESC lines with genetic abnormalities.

Since the first report of DM1-specific hESC, several studies have described the derivation of additional DM1-specific hESC lines with less than a dozen available on 1 January of 2023 at the International Human Embryonic Stem Cell Registry (www.hescreg.eu, accessed on 1 January 2023) [72,73,74]. However, the use of disease-specific hESCs presents different limitations. First, the number of disease-specific PGD embryos is limited. Importantly, the use of these cells still raises ethical questions, which may result in limited and cautious investments [75,76]. In addition, the interpretation of experimental results obtained from those cell lines can be complicated due to the absence of a clinical history of the donor.

More recently, the outstanding breakthrough of human-induced pluripotent stem cell (hiPSC) generation opened a new era for disease modeling. The generation of hiPSCs relies on the genetic conversion of a patient’s somatic cells into an embryonic-like state, lately allowing for the generation of all kinds of patient cell lines [77]. A growing number of studies have described the generation of hiPSCs derived from primary fibroblasts of DM1 patients [78,79,80,81,82]. More recently, hiPSC lines have been generated from DM1 immortalized lymphoblastic cells, opening up the possibility of using a patient’s blood samples, which are more accessible than skin biopsies [83].

At the pluripotent state, DM1-specific hPSCs have mainly been used to address questions related to the instability of CTG repeat length. On that topic, Du and colleagues first showed that CTG repeats are highly unstable during the reprogramming process and during their subsequent passages in culture. Notably, the expansion occurrence of the repeats will be enhanced if the initial CTG tract is longer [82]. A substantial increase of abnormal methylation was also observed in the hiPSCs derived from DM1 patients’ fibroblasts, indicating that aberrant methylation patterns can be re-established following reprogramming [72]. Interestingly, the instability of these CTG repeats is detected only in undifferentiated cells and not when those cells are differentiated into a specific cell type, contrasting to what is described in patients [79,82,84]. This variance could be related to the methylation status of the *DMPK* gene and the activity of mismatch repair enzymes, which have been shown to be involved in the CTG instability in mice models [85,86,87,88]. Consistent with this hypothesis, the mismatch repair components MSH2, MSH3, and MSH6 have been found to be highly expressed in DM1-specific hiPSC compared to their parental fibroblasts [82,89]. The role of MSH2 in CTG repeat stability has been recently confirmed by using DM1-specific hESCs depleted for this mismatch repair protein [90]. Regarding the epigenetic changes, a hypermethylation has been observed upstream of CTG repeats when these CTG repeats exceeded 300, which may be related to the larger CTG expansion. Notably it has only been observed in DM1 maternally-derived hESC lines, suggesting that DMPK methylation may account for the maternal bias of congenital transmission [91].

## 4. Progressive Development of Multi-Lineage hPSC-Derived Platforms for DM1

Proper disease modeling using pluripotent stem cells requires two major conditions: the possibility to differentiate into the lineages principally affected, and the ability of those derived cells to reproduce the key hallmarks of the disease. The last two decades have witnessed unprecedented advances in the capacity to direct the differentiation of hPSC into specific cell types, resulting in a substantial usage of disease-specific PSCs to address physio-pathological questions.

In the context of DM1, Mateizel et al. were the first to describe the ability to differentiate DM1 hESC into a homogenous mesenchymal progenitor cell population [92]. The presence of the key characteristics of DM1 (nuclear mutant mRNA aggregates, MBNL proteins sequestration, alternative splicing defects) validated the pathological relevance of this first DM1 hPSC-based model. Those models have been subsequently used for high-throughput drug screening and drug testing [93,94]. Since then, a growing number of studies have successfully reported the possibility of reproducing classical DM1 molecular hallmarks in more pathologically relevant cell types, but also to highlight the deregulation of new molecular pathways (Table 1).

### 4.1. Skeletal Muscle Cells

Up until now, the most efficient protocols for differentiating human pluripotent stem cells into reasonably mature muscle cells relied on the forced ectopic expression of myogenic specification factors [114]. Typically, the myogenic factor MYOD was one of the earliest examples of “master” transcription factors shown to be capable of transdifferentiating cells from one lineage (e.g., fibroblasts) into another (skeletal muscle) [115]. Based on this approach, DM1 hiPSC-derived skeletal muscle cells were generated and characterized in a couple of studies, pointing out their capacity to reproduce the main molecular features associated with DM1 [96,98]. Dastidar and colleagues also demonstrated the possibility of using these skeletal muscle cells derived from DM1 hiPSC to validate the potential of CRISPR/ Cas9 technology for the gene editing of repeat expansions [98].

Where the ectopic expression of MYOD provides a quite simple way to generate skeletal muscle cells from hPSC, this direct reprogramming approach circumvents the early developmental stages. Thus, the details of how the cells are affected during this differentiation process remains largely unclear [114]. Recently, more complex and sequential protocols, using small molecules and growth factors, have been established to better recapitulate the successive developmental stages involved in skeletal myogenesis [116,117]. DM1-specific hPSC-derived skeletal muscle cells obtained from different protocols were shown to recapitulate the main pathological hallmarks, as previously described by the primary skeletal muscle cells derived from patients [81,96,99,100,101]. Notably, the skeletal muscle cells derived from DM1-specific hiPSC and hESC both present altered myogenic fusion, as do the DM1-associated foci that sequester MBNL proteins and subsequently lead to alternative splicing defects. It is interesting to note that the depletion of MBNL proteins recapitulates these phenotypes, as recently demonstrated through the use of hiPSC depleted in MBNL proteins using CRISPR/ Cas9 technology [81]. These results strongly support the pathological contribution of MBNL proteins, but also highlight the temporal requirement of these proteins during in vitro myogenesis. Where the generation of skeletal muscular progenitors, namely myoblasts, is not affected, the late differentiation of myoblasts into multinucleated myotubes is altered by the depletion of MBNL proteins, as is also observed in DM1 [81].

As observed in DM1 patients, a recent study described a defective proliferation of hiPSC-derived satellite cells. Those cells represent the resident muscle stem cells due to their unique anatomical position at the periphery of the myofibers and their regenerative capacities [99,118]. This proliferative impairment could be associated with an abnormal induction of autophagy and defective mTOR signaling [99]. The overexpression of MBNL proteins in DM1 hiPSC-derived satellite cells normalized these defects, validating the importance and pathological role of these two pathways.

Despite these developments, several challenges remain. Notably, most of these studies have used a two-dimensional cell culture system that does not mimic the native tissue structure. Consequently, several three-dimensional models have recently started to be developed to evaluate the possibility of establishing functional skeletal muscle tissue from human pluripotent stem cells [119,120,121]. To date, only one study has successfully described the generation of 3D neuromuscular structures from DM1 hiPSC lines [113]. In this study, the authors generated mature muscle fibers thanks to a protocol allowing for the co-differentiation of hiPSC into skeletal muscle and motoneurons. They next applied this model to different neuromuscular diseases, including DM1, for which they observed abnormal calcium transient at the muscular level. In the absence of a precise characterization for the molecular hallmarks of the disease, as well as the absence of an isogenic control, the interpretation of these results is difficult. Nonetheless, this type of development clearly opens the path to more complex models that could better recapitulate the physio-pathological mechanisms.

### 4.2. Cardiomyocytes

For approximatively 80% of DM1 patients, cardiac deficiency often precedes skeletal muscle involvement and causes a high incidence of sudden death (30%) [122]. The cardiac phenotypes of DM1, characterized by electrocardiographic (ECG) abnormalities, include conduction defects, as well as ventricular and atrial arrhythmias [123]. Over the last few years, several studies have reported the generation of DM1 hiPSC-derived cardiomyocytes (CMs), thanks to the establishment of guided differentiation protocols [95,97,103,105,106]. These cardiac differentiation protocols usually lead to a heterogeneous mixture of ventricular-, atrial- and nodal-like cells. Despite this variability and the level of maturity of the cells generated, these different studies confirmed the presence of the conventional DM1 molecular hallmarks, as previously observed in heart biopsies from DM1 patients. These included the formation of aggregates of mutated DMPK mRNAs that trapped MBNL proteins and consequently altered the alternative splicing affecting the expression of *MBNL1*, *MBNL2*, *TNNT2*, *RYR2*, *ANK3*, and *SCN5A* transcripts [95,103]. A complete analysis of the molecular defects is now available, thanks to an unbiased transcriptome-wide analysis performed on DM1 IPSC-derived cardiac cells before and after the excision of the CTG expansion repeat by CRISPR/ Cas9 [98]. Since several of the alternative splice defects and differentially expressed transcripts observed in the DM1 condition are known to be involved in the electrical properties of cardiac cells, the electrophysiological characterization of DM1 hIPSC-derived cardiomyocytes has been investigated using patch-clamp recordings. These analyses revealed increased calcium currents in DM1 hiPSC-derived CMs that can be correlated with the increased expression of *CACNA1C* transcripts that encode the alpha-1 subunit of a voltage-dependent calcium channel CaV1.2 protein [95,105]. A functional correlation has also been made between the alternative splice defect of the *SCN5A* transcript, which encodes the NaV1.5 cardiac sodium channel, and alters the gating properties of this ion channel [105]. Finally, differences in the mechanical properties of DM1 hiPSC-derived CMs, including spontaneous action potential and beating, have been also detected using Atomic Force Microscopy [103,106]. One study demonstrated the possibility of normalizing these mechanical defects by treatment with anti-antiarrhythmic drugs, such as Ranolazin, opening up the possibility of testing other therapeutic options on these phenotypes [103].

### 4.3. Neural Lineage

If the involvement of the skeletal and cardiac muscles is identified early in DM1, the disease also induces important neurological manifestations [124]. The DM1 cognitive profile is characterized by multiple deficits, including intelligence, memory, language, apathy and anxiety-related disorders, and excessive daytime sleepiness [125]. Although the pathological mechanisms have been extensively studied in regards to the skeletal and cardiac muscles, whether this RNA gain-of-function mechanism can account for the neurological symptoms is still unclear. A previous analysis of brain biopsies from DM1 patients indicated that the nuclear accumulation of mutant transcripts occurs both in neuronal and non-neuronal cells [126]. However, these results are difficult to interpret, as nervous system postmortem samples may be often damaged by the end-stage manifestations of the disease. Thus, the lack of a neuronal cell model has particularly hindered efforts to study the mechanisms causing these cognitive symptoms occur. To date, few studies have tackled this question. Only a couple of studies have successfully described the generation of neural stem cells (NSC) from DM1 hPSC that all exhibited nuclear aggregates of mutant transcripts and defective alternative splicing [80,97,109]. As described for the progenitors of skeletal muscle cells, an abnormal induction of autophagy, as well as defective mTOR signaling associated with a decreased proliferative capacity, were observed in DM1 hESC-derived NSC, suggesting a common mechanism for both the neural and muscle compartments [109]. As already mentioned, the development of three-dimensional (3D) models has attracted great attention in the field of disease modeling. This is especially true in the context of brain organoids that have been shown to reproduce specific brain structures and become a pertinent model for investigating the development and mechanisms of neurological diseases [127,128]. Very recently, Morelli and colleagues successfully generated cortical organoids from DM1 hiPSC lines. In these 3D structures, and after more than 2 months of differentiation, the authors observed the alteration of excitatory synaptic signaling in glutamatergic neurons that could be rescued by Dextromethorphan hydrobromide, an antagonist of N-methyl-D-aspartic acid (NMDA). This result suggests that targeting NMDA could represent an interesting therapeutic option for cognitive impairments in DM1 patients [110]. In the near future, these more complex cell models should undeniably improve our understanding of the mechanisms by which CTG repeat expansions affect the development and the function of different sub-types of neurons, but also non-neuronal cells. Illustrating this point, a recent study based on animal models of DM1 highlighted a potential contribution of astrocytes to the physiopathology of the disease [129]. Regarding the sub-types of neurons, different studies have shown that spinal motor neurons derived from DM1 hPSC (hESC and hiPSC) reproduced the main molecular hallmarks of DM1, but also exhibited a defective neuritic outgrowth that anterogradely contributes to the NMJ defects observed in DM1 [107,111,112]. Interestingly, a preponderant role of MBNL proteins has been associated with this phenotype [111].

It is now well recognized that DM1 hPSC lines and their derivatives represent a versatile cellular platform capable of reproducing the main molecular and cellular hallmarks of the disease, and suitable for the in vitro evaluation of new therapeutic strategies.

## 5. Towards Translational Applications

Pathological analyses using hPSC have led to the identification or validation of drugs for a growing number of diseases, including spinal muscular atrophy, amyotrophic lateral sclerosis, and Wolfram Syndrome [130,131,132]. Some of these therapeutic candidates are currently under clinical trials. In the context of DM1, hPSC has become a basis for identifying and validating a large variety of therapeutic strategies, such as small molecules, small oligonucleotides, and gene-editing strategies, capable of normalizing pathological phenotypes. To date, the combination of hPSC-based drug testing with drug repositioning has led to the identification of a small molecule that has recently been evaluated in a phase II double-blind parallel-group single-center trial [132].

### 5.1. Chemical Compounds

Based on their capacity to be propagated extensively in vitro, hPSC offer the possibility of producing relevant in vitro models that are applicable to high-throughput testing for drug discovery. Despite this property, there are still few examples of the use of DM1-specific hPSC for high-throughput screenings. Maury and colleagues were the first to describe the use of DM1 hESC-derived mesenchymal stem cells to evaluate 12,089 compounds for their capacity to modulate the number of nuclear aggregates of mutant DMPK mRNAs, thanks to a high-content screening [94]. This study highlighted cardiac glycosides as capable of increasing the number of foci per nucleus, while making them smaller. Based on these elements, further investigation was performed with digoxin, the most commonly prescribed cardiac glycoside. Interestingly, digoxin was also found to be capable of normalizing different alternative splice defects, both in DM1 hES-derived mesodermal stem cells and skeletal muscle cells, and of normalizing the defective myogenesis observed in vitro [94]. However, these compounds are associated with significant side effects that have limited their evaluation in DM1 patients.

In parallel to high-throughput screenings, the combination of PSC-based drug development with drug repositioning, a strategy for identifying new uses for existing drugs, has received great expectations. This strategy has many advantages over the traditional drug discovery process, as it allows for the reduction in the duration of drug development, is low-cost, highly efficient, and minimizes the risk of failure. It is by following this strategy that the antidiabetic Metformin drug has been identified as capable of normalizing the different alternative splice defects in DM1 hES derivatives [93]. As Metformin is a well-known AMPK activator, it is of interest to note that different studies using AICAR, another AMPK activator, have also reported splicing correction in DM1 primary myoblasts and DM1 mouse models, indicating AMPK as a therapeutic target for DM1 [133,134]. These findings led to a recent completed phase II double-blind parallel-group single-center trial that suggested a promising effect of Metformin on the mobility and walking abilities of DM1 patients [132]. However, as this phase II clinical trial has been performed on a limited number of patients, the potential beneficial effect of Metformin on mobility, but also other functional parameters, is currently being further explored in two phase III clinical trials, sponsored by Tor Vergata (2018-000692-32) and Assistance Publique—Hôpitaux de Paris (NCT05532813).

More recently, and thanks to the development of DM1 hiPSC-derived brain organoids, Morelli and colleagues identified the dextromethorphan hydrobromide, an NMDA antagonist actually tested in ongoing clinical trials for the treatment of seizures and behavioral hyperactivity (NCT01520363 and NCT00593957), as capable of normalizing the progressive cortical neuron loss observed in these 3D structures. This proof-of-concept study demonstrates the potential of using cortical organoids derived from hPSC to identify new drug candidates for DM1 [110].

### 5.2. Gene Editing

The genome editing approach represents an alternative therapeutic strategy for correcting the DM1 mutation. Xia and colleagues have performed the first-proof-of-concept of this strategy in DM1 hiPSC-derived NSC [108]. Their approach consisted of introducing 2 poly(A) signals upstream of the DMPK CTG repeats by using a homologous recombination (HR) mediated by a pair of site-specific transcription activator-like effector nucleases (TALEN). This strategy led to the decreased production of expanded CUG transcripts, the diminution of nuclear RNA foci, and the reversal of aberrant splicings in neural progenitor cells derived from DM1 hiPSCs. A second study from the same laboratory repeated the experiment in DM1 hiPSCs at the pluripotent state, where intranuclear foci had already been detected [78]. They found that edited DM1-specific hiPSCs remained pluripotent, as attested by the teratoma formation, and could be successfully differentiated in neural progenitor cells and cardiomyocytes that no longer expressed tissue specific to DM1-associated splicing defects. More recently, this strategy has been modified by using CRISPR/Cas9 technology to insert the PolyA signals in the 3′ UTR region, still upstream of the expanded CTG repeats. It has been demonstrated that the SpCas 9 nickase system can produce a nick guided to a specific genome site using a sequence-specific gRNA that is preferentially repaired by homology-directed repair (HDR), which allows the insertion of PolyA signals. As a consequence, the elimination of toxic RNA CUG repeats promoted the pathological phenotype reversal of DM1 iPSCs, neural progenitors, cardiomyocytes, and skeletal muscle myofibers [97].

A second approach targeting the excision of the CTG repeat expansion has been more recently developed, thanks to the CRISPR/Cas9 technology [98]. The authors first assessed the potential of CRISPR/Cas9 in DM1 hiPSC-derived skeletal muscle cells that stably expressed the Cas9 and guided RNAs after lentiviral transduction. Genome edition led to the correction of ribonuclear foci staining and the normalization of SERCA1 exon 22 mis-splicing in the DM1 myogenic cells. Edited DM1 hiPSC lines were also generated with a non-viral gene method, using the nucleofection of ribonucleoprotein (RNP) complexes composed of Cas9 proteins and specific synthetic single guide gRNA (sgRNA). The excision of the expanded CTG repeats resulted in the disappearance of ribonuclear foci in the corrected DM1 hiPSC, DM1 hiPSC-derived skeletal muscle cells, and primary DM1 myoblasts [98]. Along this line, this method has been successfully applied to the excision of CTG expansion repeats in DM1 hiPSC-derived cardiomyocyte-like cells [135]. This resulted in the disappearance of ribonuclear foci, as well as the complete reversal of the underlying spliceopathy in DM1 cardiomyocytes, as demonstrated by an unbiased transcriptomic analysis [135].

Both approaches developed in DM1-hiPSCs demonstrated the potential of genome editing for the treatment of the disease. These different methods promoted the persistent reversion of the DM1 mutant phenotype in hiPSCs and their progenies. Further analysis would help to better characterize the ON-target and OFF-target effects associated with genome editing, such as the inversion of expanded CTG repeats induced by double strand break generation on either side of the repeats that can be revealed by a double FISH analysis, using sense and antisense oligo probes [97].

### 5.3. Antisense Oligonucleotides

In addition to the genome editing approach, one of the promising therapeutic strategies is the use of antisense oligonucleotide (ASO), that targets the DMPK transcript containing the CUG repeat expansion. Different studies have shown that ASOs have the potential to decrease mutant mRNAs aggregates, leading to the release of MBNL1 and the normalization of different defective alternative splicings in DM1 skeletal muscle cells, and in the skeletal muscle-specific murine model of DM1 [68,136,137]. However, since ASOs do not cross the blood–brain barrier after their systemic administration, and because the lack of accessibility to DM1 neurons from patients, the therapeutic potential of the ASO had not been evaluated for the treatment of neural symptoms in DM1. To explore whether those therapeutic molecules could also benefit DM1 patients after intracerebroventricular injection, Ait Benichou et al. have first evaluated the efficacy of the ASO gapmer (IONIS 486178) in neural progenitor cells derived from DM1 hiPSCs [138]. The gapmer, tested at multiple doses, exhibited a maximal efficacy on intranuclear foci reduction and DMPK mRNAs expression at 500nM in DM1 hiPSC-derived neural stem cells. This effect was correlated with a nuclear redistribution of MBNL1 and MBNL2, as well as the correction of splicing defects such as MBNL1 and MBNL2 exon 7 exclusion, APP exon 7, GRIN1 exon 4, and SORBS1 exon 23 inclusions. These in vitro pre-clinical data allowed the setting up of the evaluation of IONIS 486178 ASO potency in DMSXL mice, a systemic murine model of DM1. Following an intracerebroventricular injection in adult heterozygous DMSXL mice, the IONIS 486178 ASO induced a decrease of up to 70% in the levels of mutant DMPK mRNAs throughout different brain regions. After neonatal administration, it also reversed behavioral abnormalities, confirming its therapeutic potential to target neurological damages in DM1 [138].

## 6. Conclusions

The use of patient-derived PSCs for disease modeling and drug screening has resulted in tremendous progress over the past few years. DM1 is one of the best examples for which the full experimental paradigm of hPSC for disease modeling and drug screening has been demonstrated, leading to the identification of new therapeutic strategies.

Despite the undebatable advantages of PCS-derived cells, one should keep in mind that the comparison of animal and patient biopsies is still essential to validate the pertinence of these models. Furthermore, the observations of the physio-pathological mechanisms made in those models still need proper controls. The development of three-dimensional models has also been proposed as systems that are more relevant compared to two-dimensional cultures. Future research on in vitro human DM1 PSC-based three-dimensional models will surely allow for a better capture of the disease’s complexity. Finally, the association with “omic” technologies, such as single cell RNA sequencing, should represent a new stage of research to unravel new pathological mechanisms. The adaptation of three-dimensional models for high-throughput screening approaches might also represent an important development for the identification of new therapeutic strategies for DM1.

## Figures and Tables

**Table 1:** Summary of the different pathological phenotypes identified in DM1 hPSC and their derivatives.

DM1 Biomarker	Assay	References
Human DM1 Pluripotent Stem Cell		
CUGexp RNA Foci	RNA Fish	[78,80,95,96,97]
CTG repeat instability	PacBio sequencing, Small-pool PCR and Southern blot	[82,84,89,90]
Human DM1 Pluripotent Stem Cell-Derived Mesenchymal progenitors		
CUGexp RNA Foci	RNA Fish	[94]
Splicing defect: *INSR ex11*, *ATP2A1 ex22*	RT-PCR	[93,94]
Human DM1 Pluripotent Stem Cell-Derived Myoblasts		
CUGexp RNA Foci	RNA Fish	[81,95,96,97,98,99]
CTG repeat instability	Small-pool PCR and Southern blot	[79]
MBNL1 trapping in CUGexp RNA Foci	RNA Fish and immunostaining	[95,96,98,99,100]
Splicing defects in Myoblasts: *ATP2A1 ex22*, *NFIX ex7*	RT-PCR	[79,81,98]
Splicing defects in Myotubes: *INSR* ex11, *CAP3* ex16, *mTTIN* ex5, *MBNL1* ex5, *ATP2A1* ex11, *ZASP* ex11, *TNNT3* ex7, *NUMA1* ex16, *BIN* ex11, *MEF2C* ex2, *DMD* ex78	RT-PCR	[81,95,96,100,101]
Defect of proliferation	KI-67 immunostaining	[99]
Increased autophagy	Western blot	[99]
Decreased myogenic potential	MF20 immunostaining	[81,90,102]
Human DM1 Pluripotent Stem Cell-Derived Cardiomyocytes		
CUGexp RNA Foci	RNA Fish	[95,103,104,105]
CTG repeat instability	Small-pool PCR and Southern blot	[79]
MBNL1 trapping in CUGexp RNA Foci	RNA Fish and immunostaining	[95,105]
Splicing defects: *MBNL1* ex5, *TNNT2* ex5, *INSR* ex11, *RYR2* ex4, *SCN5A* ex6, *ANK3* ex40	RT-PCR	[79,95,103,105]
Altered nuclear morphology	Immunostaining	[103]
Action potential and conduction velocity	Patch-clamp	[103,105]
Sodium channels and Calcium current electrophysiological modifications	Patch clamp	[95,105]
Alteration of the beat impulse and duration	Atomic Force Microscopy	[103,106]
Human DM1 Pluripotent Stem Cell-Derived Neural Stem Cells and Neurons		
CUGexp RNA Foci	RNA Fish	[78,97,107,108,109]
CTG repeat instability	Small-pool PCR and Southern blot	[79]
MBNL1 trapping in CUGexp RNA Foci	RNA Fish and immunostaining	[109]
Splicing defects in Neural progenitors: *MAPT* ex2-3, *MBNL1* ex5, *MBNL2* ex5 Splicing defects in neurons: *SORBS1* ex26	RT-PCR	[78,79,97,108,109]
Reduced proliferation of neural progenitors	KI-67 immunostaining	[109]
Induction of autophagy in neural progenitors	Western blot, Immunostaining	[109]
Reduced viability of Neurons	Immunostaining	[110]
Glutamate-induced excitotoxicity in neurons	Neuronal spikes and local field potentials measured using microelectrode array	[110]
Human DM1 Pluripotent Stem Cell-Derived Motor Neurons		
CUGexp RNA Foci	RNA Fish	[107,111]
Splicing defects: *NMDAR1* ex5, *CAMKK2* ex16; *CAST* ex17	RT-PCR	[107,111,112]
Altered neurite morphology: enhanced neurite outgrowth decreased axon branching	TUJ1 or MAP2 Immunostaining	[107,111,112]
Impaired communication of spinal motor neurons with skeletal muscle targets: Acetylcholine receptor clustering Enhanced Calcium transient	Co-cultures, time lapse phase contrast microscopy, immunostaining	[111,113]

## Data Availability

Not applicable.
